# Cough Reflex Testing in Dysphagia Following Stroke: A Randomized Controlled Trial

**DOI:** 10.4021/jocmr1340w

**Published:** 2013-04-23

**Authors:** Anna Miles, Irene S.L. Zeng, Helen McLauchlan, Maggie-Lee Huckabee

**Affiliations:** a Department of Communication Disorders, The University of Canterbury, 66 Stewart St, Christchurch 8011, New Zealand; b Speech Science, School of Psychology, The University of Auckland, Private Bag 92019, Auckland 1142, New Zealand; c Centre for Clinical Research and Effective Practice, Middlemore Hospital, Private Bag 93311, Otahuhu, Auckland 1640, New Zealand; d Counties Manukau District Health Board, Middlemore Hospital, Private Bag 93311, Otahuhu, Auckland 1640, New Zealand; e Swallowing Rehabilitation Research Laboratory at the New Zealand Brain Research Institute, Department of Communication Disorders, The University of Canterbury, 66 Stewart St, Christchurch 8011, New Zealand

**Keywords:** Deglutition, Deglutition disorders, Dysphagia, Stroke care, Silent aspiration, Cough reflex testing, Pneumonia

## Abstract

**Background:**

Significant health issues and service delivery costs are associated with post-stroke pneumonia related to dysphagia. Silent aspiration is known to increase pneumonia and mortality in this population. The utility of cough reflex testing (CRT) for reducing pneumonia in acute stroke patients was the subject of this randomised, controlled trial.

**Methods:**

Patients referred for swallowing evaluation (N = 311) were assigned to either 1) a control group receiving standard evaluation or 2) an experimental group receiving standard evaluation with CRT. Participants in the experimental group were administered nebulised citric acid with test results contributing to clinical decisions. Outcomes for both groups were measured by pneumonia rates at 3 months post evaluation and other clinical indices of swallowing management.

**Results:**

Analysis of the data identified no significant differences between groups in pneumonia rate (P = 0.38) or mortality (P = 0.15). Results of CRT were shown to influence diet recommendations (P < 0.0001) and referrals for instrumental assessment (P < 0.0001).

**Conclusions:**

Despite differences in clinical management between groups, the end goal of reducing pneumonia in post stroke dysphagia was not achieved.

## Introduction

Significant health issues and service delivery costs are associated with post-stroke pneumonia related to dysphagia [1-3]. Although the development of pneumonia is known to be multi-factorial [4], silent aspiration (aspiration without a cough response) has been linked to increased prevalence of pneumonia and mortality [5, 6]. One study identified a thirteen-fold increase in risk of pneumonia if a patient was observed to silently aspirate on videofluoroscopic swallowing study (VFSS) [7]. Daniels and colleagues identified that 38% of stroke patients in their cohort aspirated, of whom 67% did not produce a cough response [8]. Splaingard and colleagues compared clinical swallowing evaluation (CSE) with VFSS. They found that the CSE only identified 42% of the aspirating patients; more concerning, 70% of patients with profound aspiration on VFSS were not identified as aspirating during their CSE [9]. The inability to detect silent aspiration on clinical assessment is a critical limitation in the assessment of dysphagia.

In patients with and without neurological conditions, significant relationships have been found between pneumonia rates and 1) reduced voluntary cough strength [10], 2) reduced laryngeal expiratory reflex (LER) [5] and 3) reduced evoked cough sensitivity [11-14]. Patients with dysphagia and pulmonary complications have significantly lower mean cough peak flow values than dysphagic patients without pulmonary complications with one study reporting a cough peak flow of lower than 242 litres/min predicting the development of pneumonia (sensitivity 77%, specificity 83%) [15]. Aviv and colleagues (1997) found increased pneumonia rates in patients post stroke with bilateral laryngopharyngeal sensory impairments [5, 16]. Nakajoh and colleagues studied the incidence of pneumonia in 143 post-stroke patients residing in a nursing home facility [11]. They found a significant relationship between pneumonia rates, delayed swallowing response relative to water injected into the pharynx and reduced evoked cough thresholds to citric acid. Patients with lower evoked cough sensitivity and slower swallowing responses were more likely to develop pneumonia. Addington and colleagues found that if a patient had a brainstem or cerebral stroke and an abnormal laryngeal cough reflex (LCR), they had a significantly higher risk of pneumonia [17]. In their study of 818 patients admitted with stroke, they found that 90% of patients had a normal LCR to tartaric acid and only 3% of this group developed pneumonia. Of the 10% with an abnormal LCR, 11% developed pneumonia. They hypothesised that the transient or permanent impairment of the LCR, irrespective of the stroke location, relates to what they term ‘brainstem shock’. They define this as a global neurological response leading to reduced consciousness, reduced respiratory drive and impaired cough reflex and comment that this needs to be addressed in the acute stages of stroke management [17].

The distinction between cough types has been well described [18, 19]. A voluntary cough is a cortically driven cough to command. A cough reflex is a three-phase process: an inspiration, followed by a forceful expiratory effort against a closed glottis, and finally the re-opening of the glottis and fast expiratory airflow [20]. A cough reflex is triggered by mechanical or chemical irritants and is often preceded by an urge-to-cough and can therefore be cortically modulated and suppressed. However it is likely that if an irritant is strong enough a pure brainstem cough reflex arc is inevitably produced without cortical control [21]. In comparison, LER is a purely brainstem driven act without cortical modulation and consists of a strong, brisk expiration without an initial inspiration suggesting a different afferent pathway from the inspiratory beginnings of a cough reflex [22]. The importance of cough type distinction is less clear in studying dysphagic patients with neurological disease where all types of cough impairment have been reported [14, 23]. Reduced strength of voluntary cough may exacerbate pulmonary consequences resulting from aspiration by inadequately clearing aspirated material from the airway [24]. The mostly likely function of the LER is the immediate protection of the airway and removal of laryngeal-penetrated material [25]. However in reality, it is usually followed by a series of cough reflexes [14, 26].

A test of evoked cough sensitivity has received recent attention as a potential adjunct to clinical swallowing assessment. A recent study by Wakasugi and colleagues validated the use of a citric acid evoked CRT paired with a water swallow test against VFSS or endoscopic study of swallowing results in 204 patients suspected of dysphagia [27]. When evaluating all 107 patients with documented aspiration, sensitivity of the CRT for detection of aspiration was 0.67, specificity was 0.97; positive predictive value was 0.98, and negative predictive value was 0.61. When the water swallow test was combined with CRT, 89.1% of those predicted to be normal were actually normal, 73.7% of those predicted to be audible aspirators actually aspirated with a cough response, and 88.2% of those predicted to silently aspirate were actually silent aspirators.

Previous research suggests that the addition of a test of cough sensitivity to CSE has the potential to reduce pneumonia after stroke [14, 27, 28]. Addington and colleagues report on the addition of a tartaric acid CRT to dysphagia evaluation of acute stroke patients by comparing two hospitals with reportedly similar clinical practice [14]. At one hospital not using CRT, 13% of participants developed pneumonia compared with 1% of participants at a comparable hospital who received a CRT. They utilised a clinical treatment algorithm for oral intake based on CRT results where a failed cough test resulted in no oral intake and a passed cough test resulted in oral intake. An algorithm with heavy reliance only on cough test findings for decision-making could be perceived as a limitation of this study as clinical management generally incorporates interpretation of other clinical indices.

A clinical guideline on the assessment of cough was produced in 2007 by the European Respiratory Society Task Force which highlighted the lack of standardisation of cough testing protocols and tussive agent dosage [20]. Morice and colleagues report capsaicin and citric acid as more stable agents and thus with more reliably consistent results, when compared to tartaric acid [29]. Until recently, no normative data were available regarding the dosage of a tussive agent that should elicit a cough response in healthy individuals. However, recent research by Monroe and colleagues [30] established normative data from a sample of 80 healthy individuals using a method of passive inhalation of citric acid through a facemask. They found that the majority (92.5%) of healthy individuals elicited a natural cough at 0.8 mol/L and that 68% also demonstrated a suppressed cough at this level; i.e. a cough evoked while the participant was actively trying to inhibit a response. At 1.2 mol/L, 80% of healthy people could no longer suppress a cough.

The most appropriate method for CRT in the dysphagic population is debatable. Addington and colleagues used a mouthpiece and an expiration-inspiration method which required the subject to exhale and then deeply inhale through the mouthpiece with the nose occluded [14] as was recommended by the European Respiratory Council (ERC) task force for most CRT situations [20]. Conversely, Wakasugi and colleagues used a passive facemask method of administration in which subjects inhaled a mist of citric acid presented by an ultrasonic nebuliser for 1-minute [27]. In comparison to the mouthpiece method, this may be a more reliable delivery method for the neurologically impaired population, where co-existing impairments of cognition, language, apraxia or oro-motor weakness (namely lip seal) are present. Addington and colleagues state that leakage around the mouthpiece and “puffing” the nebulizer were not considered effective inhalations but do not discuss how many of their acute stroke patients could not perform the task and were therefore excluded from analysis. Exclusion of the more severely impaired patients may have contributed to their low rates of pneumonia. However the two methods likely trigger different types of cough responses with the 15-second passive facemask method eliciting an evoked-cortically modulated cough and the mouthpiece method eliciting a true LER followed by a series of coughs. The use of a suppressed cough test has been suggested to represent a more true sensory reflex cough response in that cortical inhibition can no longer over-ride brainstem responses [18], thus balancing the limitations of the passive respiration method. Prior research on cough testing has failed to adequately control for type and concentration of tussive agent utilised in the test, has incorporated the test without clearly defined normative values on which to base decisions and has incorporated methods which can be difficult for patients with neurologic impairment to execute.

This study evaluated the clinical utility of a natural and suppressed CRT for reducing pneumonia in acute stroke patients using a stable tussive agent of controlled dosage and a passive facemask method appropriate for a neurologically impaired population. Recognizing that pneumonia is multi-factorial and that aspiration is not the singular issue, this study allowed greater flexibility in clinical decision making than the Addington study [14]. It was hypothesized that the addition of a cough reflex test to standard clinical swallowing evaluation would alter clinical decision-making and consequently reduce end-point pneumonia for acute stroke patients.

## Methods

### Patient selection

**Figure 1:**
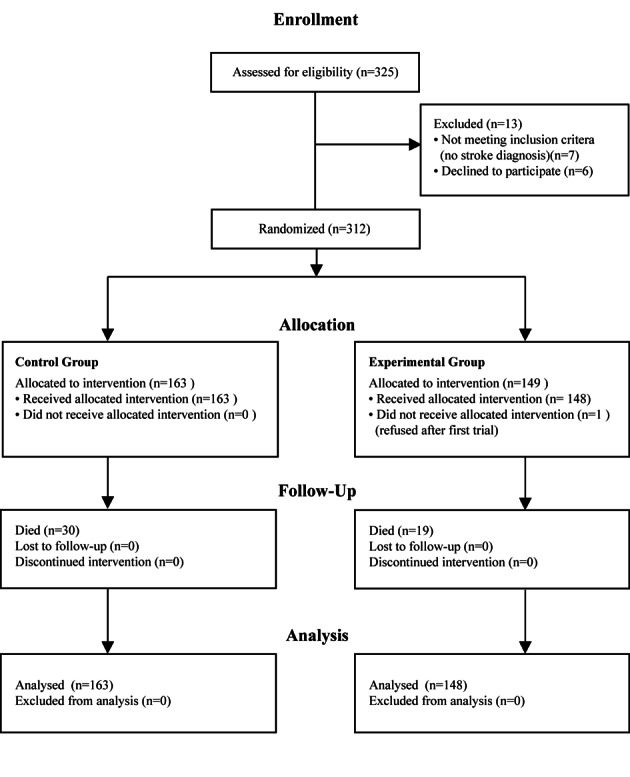
Clinical trial allocation information.

Three hundred and eleven acute stroke patients (165 females, 146 males) consecutively referred to speech-language pathology for swallowing assessment were recruited from four urban hospitals. For details of recruitment see Figure 1. Patients were excluded if palliative swallowing advice was requested rather than active treatment. These patients do not routinely receive full assessment protocols and pneumonia is not actively prevented. The participants’ ages ranged from 22 - 102 years (mean of 78 years, SD 13.5). Initial CT scans classified lesions as follows: 212 cortical, 48 subcortical, 8 brainstem, 12 cerebellar, 8 multi-level, 5 small vessel disease, and 18 with no new abnormalities detected on CT scan.

### Study design

This study received appropriate regional ethics approval and all participants gave informed consent independently or by proxy. Participants were randomly assigned to the control group or the experimental group based on a simple randomisation procedure using one computer-generated random numbers list held in the research office remote from the study environments. Participating clinicians at each research site telephoned the research office after gaining consent from each patient and were immediately given a randomisation assignment to either the control group or the experimental group. The non-blinded study design was unavoidable for patients, the ward clinicians, multi-disciplinary team and the researcher collecting the outcome data. It was essential that the results of the cough reflex test were incorporated into management decisions in order to translate to change in outcomes. Documentation of cough reflex test results in a patient’s clinical notes was therefore an integral component of the protocol. The research office providing the randomisation code was blinded to research site, clinician and any patient details.

### Protocol

For those in the control group, clinical swallowing evaluation was executed as defined by local clinical protocols. At all sites, this involved a case history, cognitive/communication screen, cranial nerve examination and observation of oral ingestion of foods and fluids. For participants in the experimental group, the standard evaluation was augmented with the inclusion of CRT prior to oral trials. Clinicians who recruited, assessed and treated the participants in the study received 8 hours of formal teaching regarding CRT procedures and interpretation, as well as the opportunity for reflective practice on a regular basis throughout the recruitment period. Protocols, procedural flow charts and management guidelines were provided.

The CRT was administered using a PulmoMate Compressor/Nebuliser (model 4650I) (DeVilbiss Healthcare LLC, Pennsylvania, US) with a predetermined free-flow output of 8 litres per minute and a restricted flow output of 6.6 litres per minute. A facemask method was used, as was described in prior research [27] and utilised in establishing normative data on which this study was based [30]. Citric acid solutions diluted in 0.9% sodium chloride were prepared by each hospital pharmacy on a weekly basis. These included a lower dose of 0.8 mol/L at which 92.5% of healthy individuals produced a natural cough (an evoked, cortically modulated cough) and a higher dose of 1.2 mol/L at which 80% of healthy individuals were no longer able to suppress a cough (a true reflex cough) [30]. Patients were told that they were participating in a cough test and they were asked to cough “if they felt the need to cough”. Initially a placebo dose of 0.9% NaCl dose without citric acid was presented to coach the participant on task completion. Presence or absence of cough during a 15 second delivery period was documented. Cough response was considered positive if two or more coughs were triggered (C2 response threshold) as recommended by the ERS Task Force [20]. The test was repeated three times at the low concentration, with a 30 second interval between each inhalation to prevent tachyphylaxis [20]. The clinician then asked the patient to “try to suppress the cough as much as you can” while the same low dose was administered. If they were able to suppress a cough at 0.8 mol/L (2 out of 3 trials), the higher concentration was administered (1.2 mol/L). The suppressed cough test was passed when participants coughed on at least 2 out of 3 trials of that dose. If the patient passed the cough test, clinicians were also asked to subjectively judge whether the cough response was strong or weak: a judgement that has been shown moderately reliable in untrained clinicians [31]. Absence of a natural cough at 0.8mol/L or the ability to suppress a cough at the higher dose was considered a failed test.

Subsequent management decisions were not prescribed, but were left to the judgment of the treating clinician. In the control group, clinicians were encouraged to follow their standard clinical decision making processes. In the experimental group, clinicians were encouraged to incorporate the combined results of the clinical swallowing evaluation and the CRT into multidisciplinary dysphagia management. Multidisciplinary information sheets and training sessions were used to support carryover of a treatment plan. Stickers were placed in patients’ clinical notes containing information for the multidisciplinary team. If the patient failed the CRT, the team was advised that s/he may not show overt signs of aspiration and would be at high risk of aspiration pneumonia. If the patient presented with a weak cough, the team was advised that the patient has a cough response, but that if the patient coughed, the cough may not be sufficient to clear aspiration. When a patient passed the CRT, the multidisciplinary team was advised that the patient was likely to show overt signs of aspiration if s/he was aspirating and was therefore at better risk of protecting the airway if they aspirated.

### Outcome measures

The primary outcome measure was the proportion of patients with confirmed pneumonia within 3 months post recruitment using the criteria described by Mann and colleagues (19) where 3 or more of the following variables constitute a diagnosis: fever (> 38 °C), productive cough with purulent sputum, abnormal respiratory examination (tachypnea (> 22/min), tachycardia, inspiratory crackles, bronchial breathing), abnormal chest radiograph, arterial hypoxemia (PO2 < 70 mmHg), and isolation of a relevant pathogen (positive gram stain and culture). Secondary patient outcome measures included length of acute hospital stay in days and the percentage of patients with readmissions for chest infection within 3 months post recruitment to the study. Clinical decision parameters were also collected, including percentage of patients with completed VFSS or fibreoptic endoscopic evaluation of swallowing (FEES) during acute admission or 3 month post evaluation (namely change in frequency in referral rates with addition of CRT) and route and type of intake at 3 month review using the Functional Oral Intake Scale (FOIS) [32]. Outcomes were assessed via phone call with patient, next-of-kin, residential care staff and/or general practitioner and a chart review at 3 months post recruitment.

### Data analysis

Statistical analyses were completed using SAS 9.3. A two-sample t-test was used to compare the mean length of stay (LOS) between the control group and experimental group; data were log transformed for analysis due to its skewness distribution. Welch’s analysis of variance for groups with unequal variance was used for comparing the diets between experimental group and control group. Chi square test was used to assess the associations between categorical data outcomes between control and experimental groups. Exact Cochran-Armitage Trend Test was used to assess the associations between mortality, pneumonia, readmission and the CRT results. Multiple logistic regressions were applied to evaluate the efficacy of the cough reflex test adjusted by confounding variables (gender, site, ACE inhibitors, cardiac co morbidities, previous stroke history, respiratory co morbidities, instrument assessment and lesion locations), and the two-way interactions including cough reflex test (CRT) and cardiac co morbidities, CRT and previous stroke history, CRT and respiratory co morbidities, CRT and lesion locations, CRT and sites.

The model selections used AIC (Akaike information criterion) as the selection criteria. Firstly the full model with all confounding factors was fit, and a backward selection with AIC as selection criteria were used to select the main effect model. The two-way interactions were then added in the main effect model one by one for the final model. All analyses were based on intention-to-treat principle. An A priori minimal sample size of 268 participants (134 per experimental group) was calculated for an estimated effect size of 0.4 at the 0.05 significance level to achieve 90% statistical power.

## Results

One hundred and sixty three patients were randomised to the control group. One hundred and forty eight patients were randomised to the experimental group. See Table 1 for a comparison of the demographics between groups. Within the experimental group, 91 passed the CRT with a strong cough (61%), 31 passed with a weak cough (21%) and 26 failed the CRT (18%).

**Table 1:** Demographic Comparison Between Experimental Group and Control Group

	Experimental Group (N = 148)	Control Group (N = 163)
Demographics		
Age	Mean 76 (SD15)	Mean 79 (SD12)
Male	78 (53%)	68 (42%)
Ethnicity		
Caucasian	111 (75%)	125 (77%)
Maori	16 (11%)	11 (7%)
Pacific Islander	13 (9%)	18 (11%)
Other	8 (5%)	9 (6%)
Hospital Site		
Hospital A	43 (29%)	49 (30%)
Hospital B	37 (25%)	33 (20%)
Hospital C	52 (35%)	62 (38%)
Hospital D	16 (11%)	19 (12%)
Comorbidites		
Previous stroke history	44 (30%)	46 (28%)
Respiratory comorbidities	15 (10%)	26 (16%)
Cardiac comorbidities	103 (70%)	116 (71%)
Site of Lesion		
Cortical	102 (69%)	110 (67%)
Subcortical	34 (23%)	34 (21%)
Other	4 (3%)	9 (6%)
NAD	8 (5%)	10 (6%)
Laterality of Lesion		
Left	69 (47%)	83 (51%)
Right	64 (43%)	60 (37%)
Other	15 (10%)	18 (11%)
Diet after initial assessment mean (se) (1-3 = non-oral feeding, 4-7 = oral diets)	4.4 (0.2)	4.1 (0.2)

### Reducing secondary complications

In the unadjusted and covariates-adjusted results, there were no significant differences in pneumonia rates between the control group and experimental group (unadjusted: control 21%, experimental 26%, X^2^ = 0.76, P = 0.38; covariate-adjusted odds ratio: 1.7 (95% C.I. 0.9, 3.0), P = 0.10). There was a non-significant trend that pneumonia was associated with an adverse response on CRT (fail 35%, weak 32%, pass 21%, Z = -1.63, P = 0.11); with odds ratios of fail vs. pass 2.0 (95% C.I. 0.8, 5.2) and weak vs. fail 1.1 (95% C.I. 0.4, 3.4). See Table 2 for a summary of outcome differences between the experimental group and control group and Table 3 for a summary of outcomes comparisons between groups adjusted by demographic and clinical factors.

**Table 2:** Outcome Comparison Between Experimental Group and Control Group

	Experimental Group	Control Group	P value
Independence on admission			0.65
Public Hospital	7 (5%)	6 (4%)	
Residential care facility	13 (9%)	19 (12%)	
Home	128(86%)	138(85%)	
Independence at 3 months post assessment			0.75
Public Hospital	12 (8%)	16 (10%)	
Residential care facility	64(43%)	74 (45%)	
Home	72(49%)	73(45%)	
Mortality	20(14%)	32(20%)	0.15
Confirmed pneumonia	38 (26%)	35 (21%)	0.38
Readmission for pneumonia	7 (5%)	4 (2%)	0.28
Diet at 3 months mean (se)	6.2 (0.1)	6.0 (0.1)	0.22
Length of stay in acute ward median (IQR)	7 (5, 12)	6 (4.5, 11.5)	0.58
Receiving an instrumental assessment	27 (18%)	12 (7%)	0.004

**Table 3:** Multiple Logistic Regressions for Outcomes Comparison Between two Interventions Groups Adjusted by Demographic and Clinical Factors

	Confirmed pneumonia odds ratio (95% confidence interval)	P value	Mortality odds ratio (95% confidence interval)	P value
Received a CRT vs. did not receive a CRT	1.7 (0.9, 3.0)	0.10	0.7 (0.4, 1.3)	0.23
Age	1.4 (1.1, 1.9)	0.01	1.7 (1.2, 2.3)	0.003
Gender (male vs. female)	1.9 (1.0, 3.5)	0.04	0.9 (0.5, 1.8)	0.76
Study Sites				
Hospital A vs. Hospital C	0.6 (0.3, 1.2)	0.17	0.7 (0.3, 1.5)	0.33
Hospital B vs. Hospital C	0.4 (0.2, 0.9)	0.03	0.8 (0.3, 1.8)	0.51
Hospital D vs. Hospital C	0.3 (0.09, 0.8)	0.02	0.2 (0.05, 1.1)	0.06
ACE inhibitors (not taking vs. taking)	1.6 (0.9, 3.1)	0.13	2.7 (1.2, 5.8)	0.01
Comorbities				
Cardiac Respiratory	3.0 (1.4, 6.5)	0.01	1.9 (0.9, 4.2)	0.12
Respiratory Previous stroke	4.3 (2.0, 9.1)	2.0E-4	0.6 (0.2, 1.6)	0.29
Previous stroke	1.8 (1.0, 3.3)	0.07	1.4 (0.7, 2.8)	0.35
Site of lesion				
NAD vs. subcortical	0.9 (0.2, 3.7)	0.88	1.5 (0.3, 7.6)	0.62
Other vs. subcortical	3.1 (0.7, 13.2)	0.12	1.3 (0.2, 8.5)	0.77
Cortical vs. subcortical	0.81 (0.4, 1.7)	0.56	1.9 (0.8, 4.6)	0.17
Interaction between CRT and respiratory comorbidities		0.04		

In the unadjusted and covariates-adjusted results, there were no significant differences in mortality rates in the experimental group compared with the control group (unadjusted: control 14%, experimental 20%, X^2^ = 2.1, P = 0.15; covariate-adjusted odds ratio: 0.7 (95% C.I. 0.4, 1.3), P = 0.23). There was a non-significant trend that lower mortality was associated with the adverse response on CRT (fail 23%, weak 16%, pass 10%, Z = -1.8, P = 0.07) with odds ratio of fail vs. pass 2.7 (95% C.I. 0.9, 8.5) and weak vs. fail 1.6 (95% C.I. 0.4, 5.8). There were no significant differences in length of stay, independence on admission or at 3 months and pneumonia re-admissions to hospital between the two study groups (Table 2, 3).

**Figure 2:**
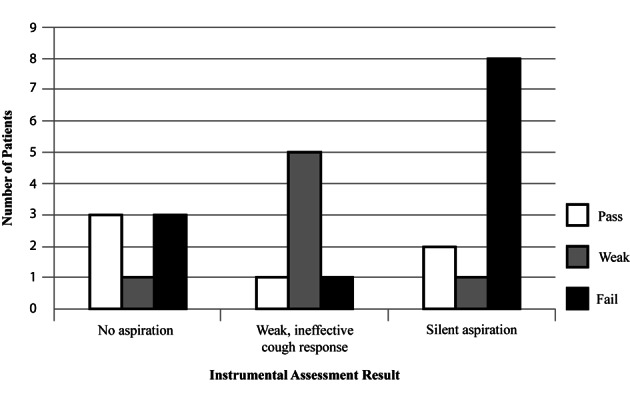
Relationship between cough test result and instrumental assessment result.

There was a significant difference in the incidence of respiratory comorbidities across CRT results (fail 8%, weak 23%, pass 7%, X^2^ = 6.7, P = 0.04). The numbers of referrals for instrumental assessment were too small for statistical analysis but descriptive analysis suggests an positive association between 1) failed CRT result and silent aspiration and 2) weak CRT result and weak response to aspiration (Fig. 2).

### Changes in clinical practice

CRT results were associated with diet recommendations following initial swallowing assessment. Higher scores on the 7 point FOIS [32] were associated with better cough test results with a mean score of 2.8 for the fail group, 3.3 for the weak group and 4.8 for the pass group (F = 13.8, P < 0.0001, mean difference and its 95% confidence interval of fail vs. pass is -2.0 (-1.1, -2.9), weak vs. pass is -1.5 (-0.68, -2.3), where a score of 1 = no oral intake, 2-3 = non-oral plus oral diet and 4-7 = total oral diets.

Overall, referral rates for instrumental assessment were low at 12%. The experimental group had a significantly higher proportion of patients referred for instrumental assessment than the control group (experimental 18%, control 7% with a proportion difference of 10.4% with 95% C.I. 2.9%, 17.9%, P = 0.004). The stronger responses to the CRT test were positively associated with a higher rate of referral (pass 8%, weak 26%, fail 46%, P < 0.0001). The numbers of referrals for instrumental assessment were too small for further statistical analysis but descriptive comparisons reveal a median number of days between cough test and instrumental assessment of 8.5 days in the control group compared with 3 days in the experimental group (pass 11, weak 2.5, fail 3).

**Figure 3:**
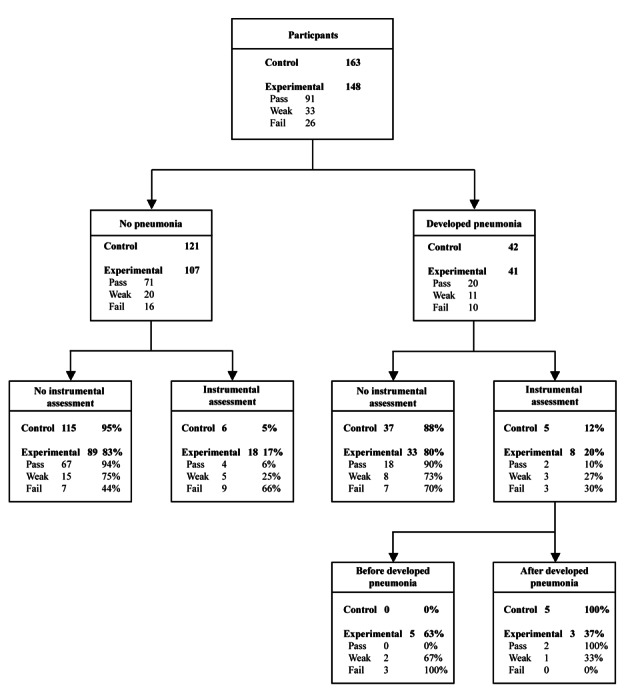
Association between development of pneumonia and instrumental assessment referral and timing.

There was a significant association between instrumental assessment and pneumonia in the control group, Χ^2^ = 3.977, P = 0.046 with an odds ratio of developing pneumonia of 3.194 (95% C.I. 0.854, 12.110). Whereas in the experimental group, there was no significant association, Χ^2^ = 0.523, P = 0.470 with an odds ratio of 1.39 (95% C.I. 0.516, 3.697) (odds ratios for pass group 2.956 (95% C.I. 0.467, 17.921), weak group 1.125 (95% C.I. 0.157, 7.828) and fail group 0.333 (95% C.I. 0.044, 2.306). Although there was still a surprisingly low referral rate of 46% for the fail CRT group and only 30% of those who developed pneumonia received an instrumental assessment, the odds ratio for developing pneumonia if a patient failed the cough test and had an instrumental assessment was reduced in comparison to other groups. Unlike all other groups, there was a difference in instrumental assessment referral rates in patients of the failed CRT group who did not develop pneumonia (no instrumental assessment 44%, instrumental assessment 66%) compared with those who developed pneumonia (no instrumental assessment 70%, instrumental assessment 30%) (Fig. 3).

The timing of instrumental assessment differed depending on CRT result again suggesting changes in clinical decision-making. The five patients in the control group and two patients in the pass CRT group who received an instrumental assessment and developed pneumonia, developed this complication prior to instrumental assessment (Fig. 3). Whereas the three patients in the weak and three patients in the failed CRT groups who received an instrumental assessment, developed their pneumonia after their instrumental assessment.

## Discussion

This study assessed the utility of CRT for changing functional outcomes in patients with dysphagia following stroke. The CRT used a stable tussive agent of controlled dosage and a passive facemask method appropriate for a neurologically impaired population. The test involved a citric acid evoked cough test - a suppressed cough test that was hypothesised to represent a true reflex cough - and a subjective test of cough strength [18, 33]. Based on prior research [14, 27], we hypothesized that inclusion of CRT in a clinical swallowing assessment protocol would provide clinicians with critical information that would subsequently alter management plans sufficiently to reduce the end-outcome of pneumonia. Our data suggest that clinicians did integrate information into diet selection as well as frequency, speed and timing of referrals for instrumental assessment. However, incorporation of the test results in multidisciplinary practice was insufficient to change patient health outcomes.

The development of pneumonia is multi-factorial. Langmore summarized that risk factors for developing pneumonia included dependency for feeding, dependency for oral care, number of decayed teeth, tube feeding, more than one medical diagnosis, number of medications, and smoking [4]. A single change in the course of a patient’s assessment may not to be powerful enough to alter this primary endpoint unless that change strictly controls a host of consequent management practices. Addington and colleagues did find a difference in pneumonia rates between their hospital with CRT and their ‘control’ hospital without CRT, but there is significant bias introduced to their study. By using the mouthpiece method of delivery, they likely limited participation to patients who were able to form sufficient lip seal, control respiration and follow instructions, perhaps excluding those most at risk of pneumonia from the experimental group. In using a sister hospital for comparison, control for geographical differences in patients and control of clinical practices is compromised.

Other studies have documented reduced pneumonia rates by the simple addition of dysphagia screening protocols [34] and clinical dysphagia pathways [35]. In both of these studies however, a variety of assessment tasks were introduced and subsequent clinical decisions were strictly controlled. Hinchley and colleagues collected data at 15 institutions and found positive outcomes for patients who received formal swallowing screens [34]. The screens included clear actions depending on the response to each task in the screen. Odderson and colleagues introduced an initial swallowing screen and found a reduction in pneumonia compared to prior to its introduction [35]. However the screen covered a range of criteria (following commands, voice, cough, water test, secretion management) and was again accompanied by a strict clinical decision pathway.

The complexity of changing clinical practice at individual or group levels is well documented [36, 37]. In our study, we acknowledged that development of pneumonia is dependent on a number of factors and we chose to allow individual clinicians to integrate this new information from the cough test into their existing decision making construct. However in doing so it allowed for significantly greater degrees of freedom in leading to final outcomes: that of clinician skill and choice. This may have resulted in poorer outcomes in our patient cohort. Dictating clinical behavior due to one variable may be short sighted and not represents the whole clinical picture. Perhaps developing is protocols that account for many variables that influence decisions may be more effective in reducing the secondary complications.

The outcome of those who passed the CRT was as poor as for those in the control group (pneumonia rates: control group 21%, pass group 21%). This may suggest an over-reliance on the result of the CRT in clinical decision-making, perhaps overlooking other assessment findings. The CRT is a simple test of respiratory sensitivity to an irritant and does not answer clinical questions regarding dysphagia severity. Only eight percent of those who passed the cough test received an instrumental assessment and they waited a median of 11 days for the referral. The development of pneumonia preceded all of these referrals. This suggests that the majority of management decisions were being made without the diagnostic information of an instrumental assessment and that further assessment was only being initiated in response to chest deterioration. It is important to acknowledge that Wagasugi and colleagues (2005) found a sensitivity of 67% when comparing CRT to instrumental assessment [27]. A passing response on cough reflex testing must be taken alongside all other assessment findings and not be mistaken for an instrumental/diagnostic test result. Further research is needed to explore the sensitivity and specificity of the CRT.

In the control group and passed cough test group, clinicians were using pneumonia as a clinical indicator of aspiration and only referred for diagnostic assessment when pneumonia developed. In view of the increased risk of mortality and increased length of stay associated with the development of pneumonia after stroke, this practice in clinical reasoning is of serious concern [38]. In the control group, where instrumental assessment was only completed in response to pneumonia, the odds of developing pneumonia if you had an instrumental assessment were 3.19 times higher than if you did not have an instrumental assessment. Whereas in the failed cough test group, although only 46% of patients were referred for instrumental assessment, it appears that different clinical decisions were being made. Instrumental assessment occurred earlier and without waiting for the development of pneumonia, i.e. in response to the CRT result not pneumonia development. Clinical outcomes may also look more positive in response to this practice. The odds ratios for developing pneumonia if a patient was referred for instrumental assessment was lower and 66% of the patients who did not develop pneumonia had received an instrumental assessment compared with only a 30% referral rate in those who developed pneumonia.

There are a number of limitations to this study. The use of more controlled management protocols including a validated CSE and a strict clinical pathway may have led to a clearer reduction in pneumonia rates. All clinicians received the same training, followed the same CRT protocol and were given the same guidelines for integration of the CRT results into management decisions but followed local policies and procedures. Reliability of clinician assessment, interpretation and action is uncertain.

The experimental group and control group did not differ in age, length of stay, comorbidities, stroke type or site or initial diet recommendation. This strongly suggests homogeneity between groups but the addition of an accepted stroke severity of handicap measure would have added to this comparison and to the overall interpretation of clinical outcomes. The primary and secondary outcome measures were gathered by phone and chart review, therefore running a risk of recall and surveillance bias. Information was gathered from GP, residential care staff, patient, family and clinical records in an attempt to reduce this risk.

There is great interest in the development of a bedside test for ‘silent aspiration’ in the field of acute dysphagia management. In this study, knowledge of cough response did not decrease pneumonia rates, suggesting that inclusion of a CRT test alone is not sufficient to change clinical outcomes. The findings that that negative CRT outcomes were associated with higher risk of mortality and pneumonia did not reach statistical significance but are clinically significant. Management was not controlled and integration of CRT into a clinical pathway that also controls for the other known predictors of pneumonia and stipulates the clinical decisions of clinicians may prove more successful. Further research into the validation of this clinical tool would add value to the clinical field. More importantly integration of information into clinical decision-making and clinical pathways, is warranted.

### Conclusions

Despite differences in clinical management following the introduction of a CRT, the end goal of reducing pneumonia in post stroke dysphagia was not achieved. The CRT in isolation did not change outcomes in a clinical environment where management was not controlled. Further research is needed to validate the CRT against recognized instrumental assessment tools. Investigating the integration of the results of the CRT into multi-disciplinary clinical decision-making is of interest.

## Clinical Trial Registration Information

Australian and New Zealand Clinical Trials Registry: URL; htt://www.ANZCTR9000984291.aspx, UIN; ACTRN12609000984291.
